# Functionalisation Effects on Mechanical, Electrical and Thermal Properties of 3D-Printed MWCNT/ABS Nanocomposites

**DOI:** 10.3390/polym17172428

**Published:** 2025-09-08

**Authors:** Nima Zohdi, Phan Quoc Khang Nguyen, Yixia (Sarah) Zhang, Richard (Chunhui) Yang

**Affiliations:** Centre for Advanced Manufacturing Technology, School of Engineering, Design and Built Environment, Western Sydney University, Sydney, NSW 2751, Australia; nima.zohdi@westernsydney.edu.au (N.Z.); 19146179@student.westernsydney.edu.au (P.Q.K.N.); sarah.zhang@westernsydney.edu.au (Y.Z.)

**Keywords:** additive manufacturing, fused filament fabrication (FFF), functionalisation, multi-walled carbon nanotubes (MWCNTs), acrylonitrile butadiene styrene (ABS), nanocomposites

## Abstract

While fused filament fabrication (FFF) has gained widespread popularity in additive manufacturing, its prevalent limitation in mechanical properties has prompted researchers to explore innovative solutions, with the creation of nanocomposites emerging as a promising solution. In this study, the effect of multi-walled carbon nanotubes (MWCNTs) on the material properties and morphology of acrylonitrile butadiene styrene (ABS)-based nanocomposites at various MWCNT concentrations of 0.1–1.5% is investigated. A 0.5% MWCNT addition was found to be the optimal content for mechanical, electrical, and thermal properties for FFF-printed specimens printed at longitudinal and transverse build orientations with profound improvement compared to pure ABS. Morphological analysis confirms the significant influence of air voids, low interlayer bonding and the agglomeration of additives on the properties of FFF-printed parts. Then, functionalisation methods are developed in this study for the effective modification of nanoadditives, and their influences on mechanical, electrical and thermal properties of FFF-printed nanocomposite parts are investigated. Both the covalent and non-covalent methods of functionalisation result in a uniform dispersion of nanoadditives with a positive impact on the material properties of those parts, especially for those printed at transverse build orientations.

## 1. Introduction

Additive manufacturing (AM), also referred to as 3D printing (3DP), rapid prototyping (RP), or solid freedom (SF), represents an advanced manufacturing technique wherein successive layers of materials are printed on top of one another [1,2]. The growing prevalence of AM applications can be attributed to its distinctive advantages, such as the ability to fabricate intricate geometries with high precision, optimise material usage, offer design flexibility, and enable personalised customisation. These attributes set it apart from traditional manufacturing processes [2]. Despite these contributions, AM faces challenges such as size limitations, microstructural imperfections, high equipment costs, slow printing processes, inferior mechanical properties, and anisotropic behaviour, hindering mass production efforts [2,3,4].

Fused filament fabrication (FFF), also well known as deposition modelling (FDM), is a widely utilised additive manufacturing technique that involves layer-by-layer deposition of thermoplastic materials to create three-dimensional objects. One of the key advantages of FFF lies in its simplicity and versatility, allowing for the fabrication of complex geometries with ease [5]. However, current drawbacks include limitations in material properties and mechanical strength, as well as challenges associated with recycling and sustainability in the context of conventional thermoplastic filaments [6]. There are two key approaches used by researchers in the literature to enhance the mechanical properties of FFF-printed parts: incorporating additives into the filaments to develop composite materials [7,8] and optimising process parameters [9,10,11]. Nanocomposites, which involve integrating nanoparticles, e.g., carbon nanotubes, could significantly improve strength, stiffness, and thermal stability, thereby elevating the overall mechanical performance of FFF-printed products [12].

Acrylonitrile butadiene styrene (ABS), a commonly used thermoplastic polymer, is known for its favourable properties such as durability, impact resistance, and ease of processing, making it a popular choice in FFF. The incorporation of multi-walled carbon nanotubes (MWCNTs) into ABS matrices can potentially increase the thermal conductivity, electrical conductivity and mechanical (thermo-electro-mechanical) properties, opening avenues for improved performance and functionality in FFF applications [13,14,15]. The reinforcement of 6% MWCNTs led to enhancement in the tensile modulus for all 3D-printed MWCNT/ABS specimens at various build orientations, while thermal conductivity was enhanced by 55% for specimens printed at a longitudinal orientation [16]. After evaluating the filament properties of MWCNT/ABS specimens at various nanofiller contents from 1% to 8%, Dul et al. [17] concluded that 6% was the optimal content for MWCNTs and then they fabricated 3D-printed specimens at different raster angles and build orientations for material characterisation. The presence of MWCNTs was revealed to improve thermo-electro-mechanical properties, though the poor tensile properties of transversely printed specimens remained an issue. In another study by the same authors, the inclusion of 10% MWCNTs resulted in the highest conductivity and the best tensile performance for MWCNT/ABS nanocomposites, though the effect of the 3D printing method was confirmed to strongly influence the properties compared to the filament properties [14]. Despite the extensive studies on MWCNT/ABS nanocomposites, there were no attempts to investigate and optimise the nanoadditive contents for multi-optimising of the thermo-electro-mechanical properties of 3D-printed MWCNT/ABS parts. In addition, although the nanoadditive content was optimised at the filament stage [14,16,17], the critical effects of material anisotropy [18] on 3D-printed parts with different build orientations and various additive contents, as well as their morphology behaviours, are still not fully understood.

Apart from ABS, MWCNTs were also successfully used to reinforce polymer matrices including polyetheretherketone (PEEK) [19], polylactic acid (PLA) [20], poly(methyl methacrylate) (PMMA) [15] and thermoplastic polyurethane (TPU) [21]. Although successful nanocomposites were prepared and evaluated, the agglomeration of MWCNTs remained a main issue that strongly impacted the properties [22,23], necessitating methods for ensuring uniform dispersion. While incorporating MWCNTs can lead to improvements in material properties, the melt flow index and rheological properties can be significantly reduced at high concentrations of additives [13], leading to nozzle clogging during the printing process and thus requiring the pre-optimisation of temperatures [22,24].

Despite several studies on MWCNT/ABS nanocomposites, including both filament characterisation and printed part evaluation [14], most prior work has focused on relatively high nanoparticle loadings (5–10%), where agglomeration and processing challenges become more severe. The mechanical anisotropy that is inherent to FFF, strongly influenced by build direction and interlayer bonding, has been reported [25], but its combined effect with nanoadditive concentration remains insufficiently explored. Furthermore, although the functionalisation of nanotubes is recognised to improve dispersion and interfacial bonding, direct comparisons between covalent and non-covalent strategies in the context of 3D-printed ABS/MWCNTs are scarce [26,27]. Importantly, no prior studies have attempted to determine an optimal additive concentration that balances tensile strength, electrical conductivity, and thermal conductivity simultaneously in FFF-printed ABS/MWCNT composites. The present study addresses these gaps by systematically evaluating additive content, build orientation, and functionalisation strategy together, thereby advancing the understanding of how to optimise thermo-electro-mechanical performance in printed nanocomposites.

This paper aims to (a) determine the optimal MWCNT content by evaluating the tensile strength, electrical conductivity, thermal conductivity, and morphological behaviours of FFF-printed MWCNT/ABS, and to (b) develop an effective functionalisation method for the MWCNT and evaluate the effects of the functionalisation methods on the material properties of FFF-printed parts. This paper is structured as follows: Section 2 focuses on the development of the experimental procedure including material selection, the treatment of MWCNTs using both covalent and non-covalent functionalisation approaches, the fabrication of MWCNT/ABS nanocomposite pellets, the extrusion of MWCNT/ABS nanocomposite filaments and then the 3D printing of MWCNT/ABS nanocomposite samples using FFF; Section 3 presents the obtained results and conducts the discussion; and Section 4 draws conclusions for the research.

## 2. Materials and Methods

**Figure 1:**
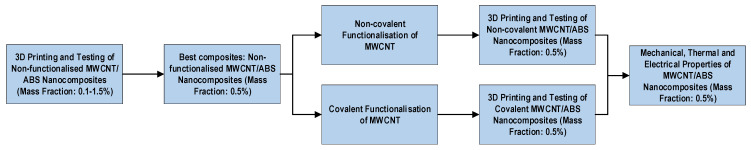
Design of Experiment.

Overall, the experimental work conducted in this research includes the 3D printing and testing of the nanocomposites with variant mass fractions of multi-wall carbon nanotubes (MWCNT) to determine the best nanocomposites and then the experimental work is further conducted with the implementation of both non-covalent and covalent functionalisation approaches of multi-wall carbon nanotubes on these nanocomposites to investigate their mechanical, thermal, and electrical properties as well as the influences of two different functionalisation methods on them. The detailed procedure is summarised in the flowchart depicted in Figure 1.

### 2.1. Materials

The polymers were obtained in the form of pellets of ABS-grade PA747 (C_8_H_8_·C_4_H_6_·C_3_H_3_N)_n_ purchased from ChiMei Corporation (Tainan City, Taiwan). The commercialised multi-wall carbon nanotubes (MWCNTs) with a diameter range of 10–15 nm and length of 5–15 µm were purchased from Nanostructured & Amorphouse Materials, Inc., Houston, TX, USA.

### 2.2. Functionalisation Methods for Modification of Nanoadditives

#### 2.2.1. Covalent Functionalisation of Nanoadditives

**Figure 2:**
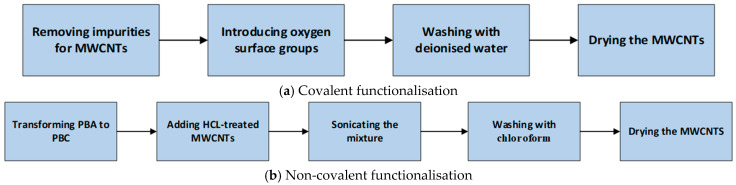
The processes of covalent (**a**) and non-covalent (**b**) modification of MWCNTs.

The method used for covalent functionalisation is based on the technique used by other researchers [26,28,29] as shown in Figure 2a.

To remove any possible impurities, nanoadditives were washed with HCL acid (37% from Scharlau) through proper mixing using a Bandelin Sonopuls HD 4050 Ultrasonic homogenizer (Berlin, Germany) in 2 s intervals and at 100% intensity for a total of 2 h. The mixture was then washed several times with deionised water to remove any possible impurities. After adjusting the PH to neutral, nanoadditives were filtered with a 1 µm DURAPORE PVDF filter and then dried in a convection oven overnight.

For covalent functionalisation, the first step to introducing oxygen surface groups is to facilitate the attack of the nitronium ion NO_2_^+^ on the carbonaceous surface. To achieve this, the nanoadditives were sonicated in a concentrated mixture of sulfuric acid (98% purity from RCI premium, Parsippany, NJ, USA) and nitric acid (70% purity from CSA scientific, Gillman (Adelaide), SA, Australia) with a volume ratio of 3:1 at room temperature for 2 h [30,31]. To prevent overheating the solution and causing unwanted damage to the structure of the nanoadditives, the container was immersed in an ice and water container. The modified nanoadditives were then washed several times with deionised water and filtered using PVDF filters until the PH of the filtered water was raised to neutral. The extracted nanoadditives were dried in a conventional oven at 65 °C overnight to remove moisture before mixing with the polymer.

#### 2.2.2. Non-Covalent Functionalisation of Nanoadditives

The MWCNTs were treated with a non-covalent functionalisation process as shown in Figure 2b. The process was carried out by first transforming 1-pyrenebutyric acid (PBA) to 1-pyrenebutyric chloride (PBC) [32,33,34]. To produce PBC, 5 g of PBA was stirred for 12 h at 30 °C in a Heidolph Hei-VAP Value rotary evaporator (Heidolph Instruments, Schwabach, Bavaria, Germany) with 1000 mL of thionyl chloride and 100 mL of DMF at 70 °C for 12 h under nitrogen gas. The remaining thionyl chloride was dried in a vacuum chamber at room temperature. The remaining paste was then mixed with 10 g of ODA followed by stirring at 100 °C for 48 h and then washing with chloroform several times until the solvent was fully removed from the system. The process resulted in brown paste alkyl amide-modified PBA (PBC), which quickly turns into a flaky light-yellow powder upon losing chloroform. PBC-m-MWCNTs were then produced by adding 5 g of HCL-treated MWCNTs to 5 g of the PBC and sonicating the mixture in chloroform for 20 h. The PBC-m-MWCNTs were washed with chloroform several times and then dried overnight in a vacuum oven at 100 °C.

### 2.3. Preparation of Polymer Nanocomposites

A heavy-duty Cole-Parmer overhead stirrer (Cole-Parmer, Vernon Hills, IL, USA) was employed to first mix the polymer (ABS) and acetone for 2 h while the system was exposed to 70 °C heated by a heating mantle. When the polymer pellets were fully melted, those treated nanoadditives were mixed with the melted polymer for another two hours in pure acetone. The viscus paste was then spread on an aluminium foil sheet and left at ambient temperature to remove acetone for two days. To further dry and remove acetone from the system, a vacuum oven was used to heat the sheet to 80 °C while the sheet was under vacuum for 12 h. Flake-sized particles were obtained by feeding the dried sheet to a crusher. The flakes were dried again to remove all the possible trapped acetone and moisture in a convection oven at 65 °C for an extra day before being fed into the extruder. It should be noted that acetone was first added to partially dissolve and swell the ABS pellets, thereby reducing viscosity and enhancing chain mobility, which facilitates subsequent nanofiller incorporation [32]. A second addition of acetone provided a common-solvent environment for co-mixing ABS and CNTs, enabling improved nanofiller dispersion prior to solvent removal [33,34].

**Figure 3:**
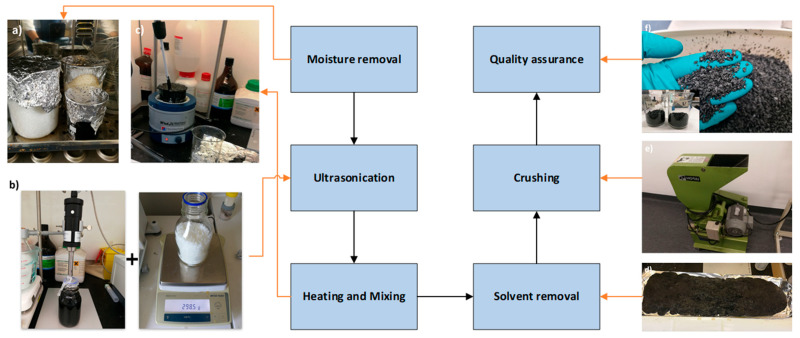
The procedure for making nanocomposites. (**a**) Moisture removal stage from polymers and additives in an oven; (**b**) ultrasonication of additives in solvent and calculating the mass ratio of the polymer/additive; (**c**) heating and mixing stage; (**d**) solvent removal stage under fume hood; (**e**) crushing stage; and (**f**) final product quality check before adding to the extruder.

Figure 3 shows the process of polymer composite production carried out in this experiment.

### 2.4. Extrusion of Pellet and Nanocomposite Filament Production

MWCNTs at different weight ratios of 0.1%, 0.5%, 1% and 1.5% were added to ABS polymers as our preliminary studies found MWCNTs exceeding 2% would lead to failure of filament extrusion. Each batch was fully dried in an oven and crushed with a crusher into flakes for the extrusion process. With the assistance of an extruder and a filament winder, the filaments of nanocomposites with a diameter of 1.75 ± 0.1 mm were extruded. The surface of the filaments was compared using scanning electron microscopy (SEM) images to understand the effect of the additives on the structural integrity of the polymer nanocomposites. To achieve a clear cut, each filament was submerged in liquid nitrogen for more than 5 min to make it brittle enough before cutting with a surgical blade.

**Figure 4:**
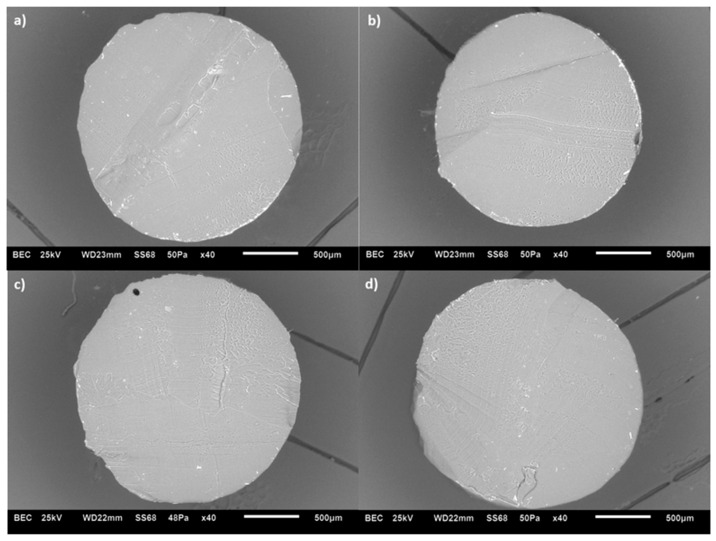
SEM images of (**a**–**d**) MWCNT/ABS at 0.1%, 0.5%, 1% and 1.5%.

Adding nanoadditives has a particular influence on the structures of the polymer nanocomposites. Figure 4 shows SEM cross-sections of extruded ABS/MWCNT filaments at different additive concentrations. At the macroscopic level, no dramatic differences were observed between samples. However, closer inspection suggests subtle variations in surface texture and the presence of irregularities that may be linked to increased porosity with higher CNT content. To some extent, only visual assessment is insufficient to quantify porosity. The structure of the air voids will be studied more in detail in the following sections to achieve a better understanding of the effect of these air voids on the thermo-electro-mechanical properties of the FFF-printed specimens.

### 2.5. Fabrication of Specimens

For tensile testing and analysis of structure, specimens of pure polymer and nanocomposites were printed in a dog-bone shape. The dimensions of Type-V specimens were adopted from the ASTM D638 standard [35]. Batches of five specimens were stacked and sliced using Prusa Slicer 2.5.0 software and then printed using a Prusa MK3S printer (Prague, Czech Republic). As the literature indicates, adding MWCNTs leads to a reduction in the melt flow index [19,36], and temperatures were pre-optimised before mass production. Based on these findings, the printing temperatures were empirically pre-optimised during preliminary trials to ensure continuous extrusion. In addition, lowering the printing temperatures below 260 °C caused nozzle clogging, which is a common phenomenon when adding additives [24,37]. Key FFF printing process parameters used for printing specimens can be found in Table 1.

### 2.6. Electrical, Thermal and Mechanical Testing

To measure the thermal properties of the FFF-printed specimens, thermogravimetric (TGA) and differential scanning (DSC) measurements were performed using an STA 449 F3 Jupiter TGA-DSC analyser (NETZSCH, Gebrüder, Germany) with closed aluminium pans. For measurement, the weights of specimens were tried to be kept almost constant at around 8 mg ± 0.5 mg (balance accuracy 0.0001). Specimens were heated from 30 °C to 590 °C at a rate of 10 K under a constant flow of nitrogen gas (25 mL/min, purity > 99%). 

To investigate the mechanical anisotropy, the tensile strength of the FFF-printed specimens in a longitudinal build orientation (LBO) and a transverse build orientation (TBO) were both measured using a Universal Testing Machine (Instron 3365 with a 5-kN load cell, Instron, Norwood, MA, USA), respectively. The tensile tests were performed according to ASTM D638.

Specimens for measuring electrical conductivity were designed and printed in a spherical shape with an overall diameter of 60 mm and thickness of 3.2 mm in both longitudinal and transverse build orientations to investigate the effect of the build orientation on the electrical conductivity of the specimens. For the electrical conductivity measurements, the discs were designed to cover the inner circle (negative) and the outer metal ring (positive) of the electrical conductivity. The thickness of the specimens was measured three times across each disc using an external micrometre. The average of measurements was used to calculate the volume resistivity of printed specimens.

**Figure 5:**
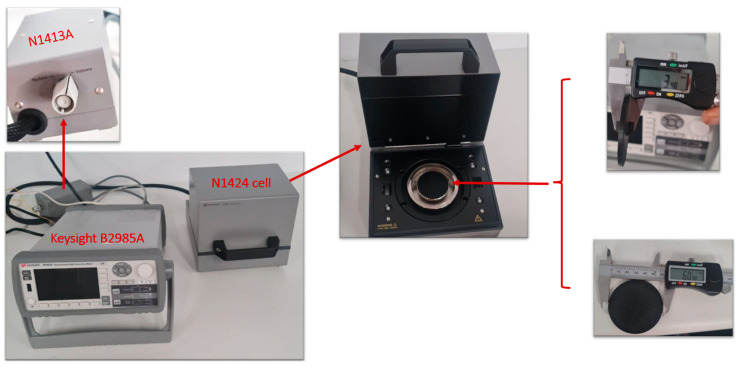
The setup for measuring the electrical conductivity.

A Keysight B2985A electrometer/high resistance meter (equipped with an N1413A high resistance meter fixture adapter and N1424A/B/C resistivity cell, Keysight Technologies, Santa Rosa, CA, USA) was used (Figure 5). Because of the interlock cell used in this setup, the instrument could hit the specimens with 1000 V to increase accuracy for both surface and volume resistivity measurements. An N1413A adapter was used to enable the use of interlock cells as well as switching effectively between surface and volume measurement. All specimens were tested at least 3 times and at each run, 10 measurements were collected to ensure the consistency and accuracy of the results.

**Figure 6:**
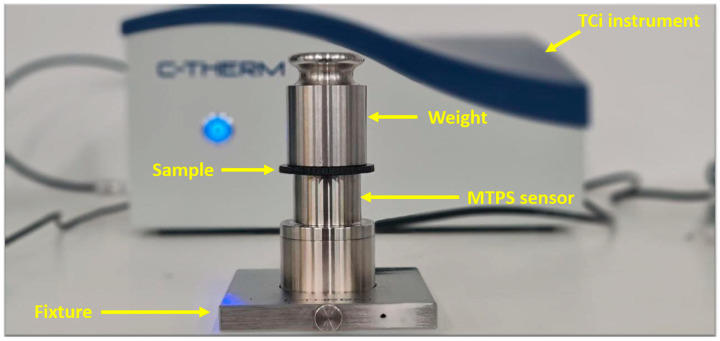
The equipment used for measuring the thermal conductivity.

For measuring the thermal conductivity of the specimens with nanoadditives, a C-Therm model TCi thermal conductivity analyser with a modified transient plane source (MTPS) sensor was used (Figure 6). The designs and dimensions of specimens used for this measurement were the same as those specimens for electrical conductivity measurements.

### 2.7. Microstructural Characterisation

For microstructural analysis, Fourier Transform Infrared Spectroscopy (FTIR) was utilised to scan nanocomposite samples and determine their chemical properties. Scanning electron microscopy (SEM) was used to distinguish nanoadditives from the polymer matrix and to determine their shape, size, morphology, orientation and distribution. Also, the layered structure and interlayer bonding of the FFF-printed nanocomposites were examined. Fourier Transform Infrared/Raman spectrometer Bruker Vertex 70 (Bruker Optics GmbH, Ettlingen, Germany) with Ram-II Module was used to confirm the existence of the functional groups on the surface of the modified nanoadditives. A 514 nm green line of argon was used to acquire the Raman spectra in a range of 1000 to 3000 cm^−1^. The FTIR spectrum was measured in a range of 400 to 4000 cm^−1^ by making the mixture of additives and KBR discs and assigning pure KBR as the background.

In this study, different scanning electron microscopy (SEM) instruments were employed to demonstrate the structural integrity and air voids produced during the 3D printing process. Specifically, the FlexSEM 1000 II (Hitachi High-Tech Corp., Tokyo, Japan) and the JEOL 6510LV SEM (JEOL Ltd., Tokyo, Japan) were used. The Ultra-Variable-Pressure Detector (UVD) and backscattered electron (BSE) features were used to record images at low-accelerating voltages and low-vacuum conditions. Specimens with clear and smooth surfaces were prepared by Leica RM2125 RTS (Leica Biosystems, Wetzlar, Germany) before placing them in the SEM instrument. To compare the dispersion quality and diameter of the additives before and after the functionalisation process, the Zeiss Merlin FEG-SEM instrument (Carl Zeiss Microscopy GmbH, Oberkochen, Germany) was used. A diluted suspension of nanoadditives was prepared by dispersing the additives in acetone followed by ultrasonication for 2 h. Suspensions were made freshly and immediately transferred on a piece of silicon wafer and left overnight before performing microscopic analysis.

## 3. Results and Discussion

### 3.1. Effect of MWCNT Content on the Properties of Non-Functionalised MWCNT/ABS Nanocomposites

#### 3.1.1. TGA

**Figure 7:**
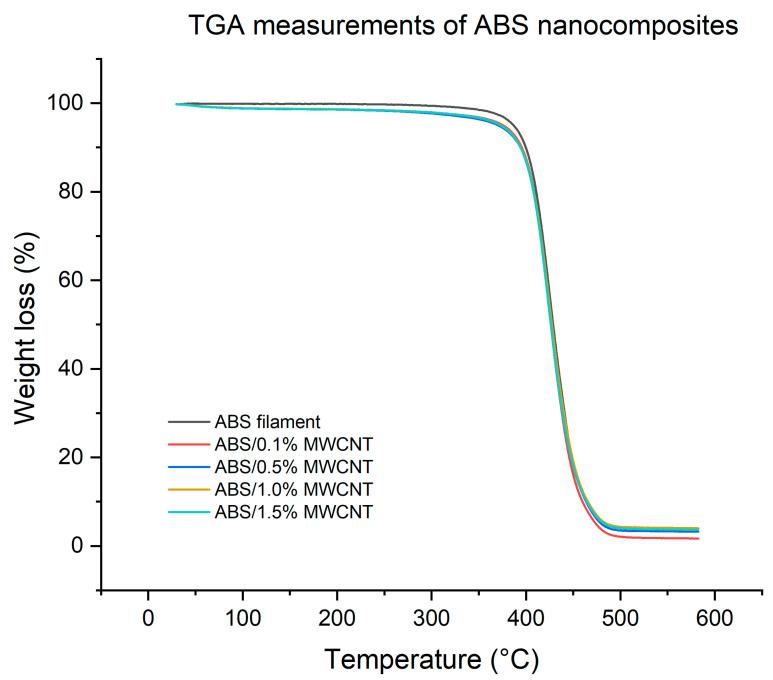
TGA results of MWCNT/ABS nanocomposites.

To explore the effect of adding nanoadditives on the thermal stability of the MWCNT/ABS nanocomposite filaments, TGA was conducted. TGA results are summarised in Table 2 and Figure 7. All the ABS-related specimens start to decompose around 407 °C to 409 °C. From the TGA result, a printing temperature range of 220 °C to 260 °C was selected as safe temperatures as the decomposition temperatures happen at almost double those printing temperatures. The decomposition temperature was determined at 2% weight loss. The incorporation of MWCNTs did not cause any significant change in decomposition temperatures, as the differences remained within experimental variation. The decomposition temperature of 408.9 °C of pure ABS is also similar to what was reported by Dul, Fambri and Pegoretti [17] (416.9 °C). In addition, residual char contents were found to be 4.0% for pure ABS and between 1.7% and 4.0% for the nanocomposites, respectively. There is a slightly higher char values for the 0.5%, 1.0%, and 1.5% samples, which is consistent with the thermal stability of CNTs at elevated temperatures. In a different system, incorporating MWCNTs from 0.5% to 5% into PLA matrix also did not show any rapid changes [22].

#### 3.1.2. DSC

**Figure 8:**
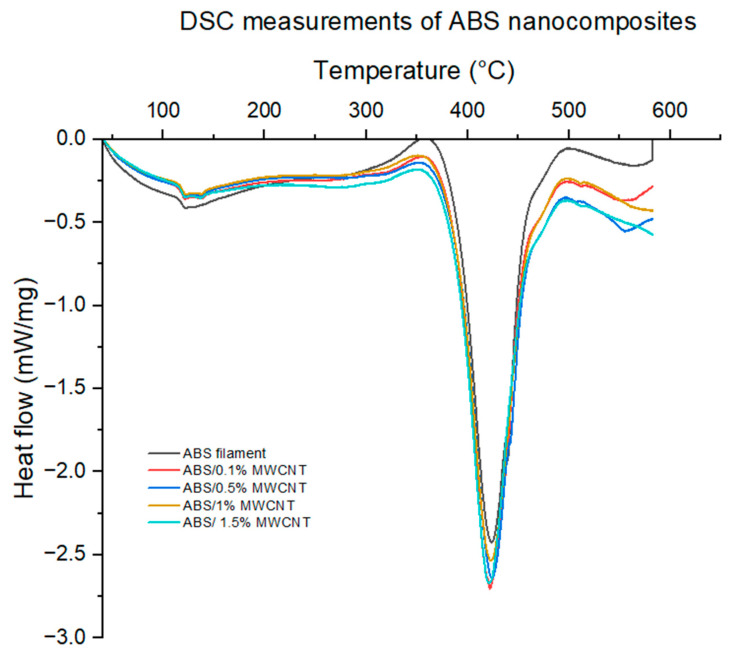
DSC results of MWCNT/ABS nanocomposites. Endothermic heat flow is plotted in the downward direction.

Table 2 and Figure 8 show the DSC results of MWCNT/ABS nanocomposites. The glass transition temperature of the pure ABS polymer and its associated nanocomposites falls within the same range for each category. DSC results confirm that the selected bed temperature of 115 °C is well above the glass transition temperature of the polymers and the polymer nanocomposites which guarantees a good adhesion between the first deposited layer and the heated bed.

#### 3.1.3. Mechanical Characterisation of Non-Functionalised MWCNT/ABS Nanocomposites

The effects of MWCNT nanoparticles on the tensile strength (TS) of pure ABS polymer and MWCNT/ABS nanocomposites in two build orientations of longitudinal (LBO) and transverse (TBO) are listed in Table 3.

**Figure 9:**
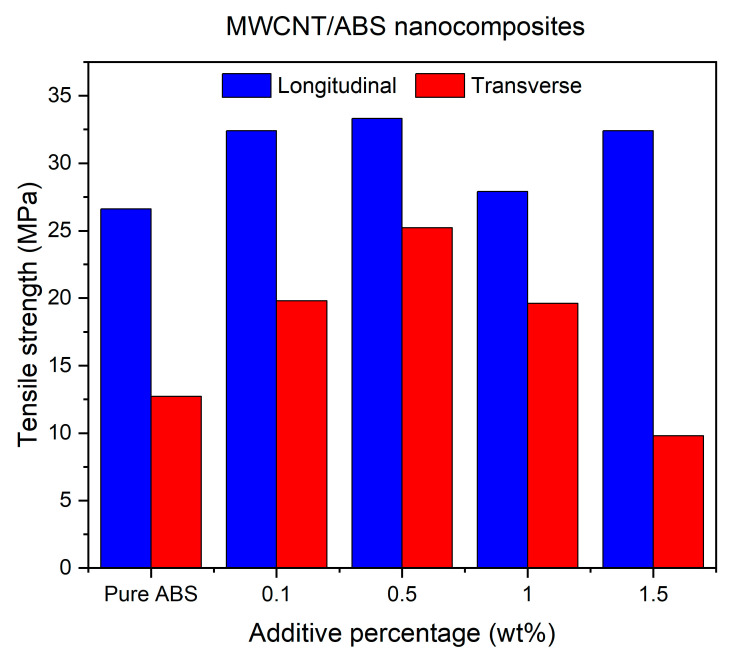
Tensile strength of MWCNT/ABS nanocomposites printed at LBO and TBO.

Figure 9 demonstrates a comparison between TS results of MWCNT/ABS nanocomposites printed at 260 °C with different weight ratios of MWCNTs and those printed at two different build orientations of LBO and TBO. Regardless of build orientation, by increasing the additive percentages, the TS values show an increase of up to 0.5% of MWCNTs in ABS polymer. With the reinforcement of MWCNTs, the load applied to the MWCNT/ABS nanocomposites is transferred to MWCNTs, contributing to an enhancement in TS. A similar phenomenon was found with MWCNT/PLA nanocomposites in the literature [38].

One notable difference is that specimens printed at LBO exhibit higher strength compared to those printed at TBO. Additionally, the lowest degree of anisotropy is observed at a 0.5% inclusion. Similar phenomena were reported for ABS/6% MWCNT nanocomposites printed at two different build orientations [16], or even when hybrid GNP/MWCNT was used for reinforcement [36]. The results reveal that incorporating the MWCNTs can have a positive influence on improving the mechanical properties of the printed specimens by up to 0.5% for both the LBO and TBO. The reason why 0.5% MWCNTs provided maximum tensile strength compared to 1% or 1.5% could be attributed to the inherent behaviour of MWCNTs to form clusters in ABS polymer at high concentrations, as confirmed to be the main issue for the reduction in TS [13,22,23]. This agglomeration can potentially increase the uneven distribution of the additives amongst the specimens. As a result, this can increase the chance of polymer chain ruptures from agglomerated spots and the premature failure of the specimen when it is under stress.

The Analysis of Variance (ANOVA) for tensile strength in Table 4 indicated that build orientation had a statistically significant effect (*p* = 0.005), whereas additive concentration alone was not significant. This outcome highlights that interlayer bonding and anisotropy remain dominant factors in the mechanical response of FFF-printed parts, which is consistent with prior studies showing that orientation effects often outweigh filler content in determining tensile performance. Nevertheless, functionalisation strategies can still improve load transfer when dispersion and interfacial adhesion are enhanced, suggesting that the combined influence of orientation and interfacial design should be considered in future optimisation [39]. While the variation in tensile strength with additive content was relatively modest, ANOVA confirmed that build orientation was the statistically significant factor, reinforcing that interlayer bonding and anisotropy exert a stronger influence than filler concentration alone on the mechanical response of FFF-printed parts.

#### 3.1.4. Morphology Analysis of 3D-Printed Specimens

**Figure 10:**
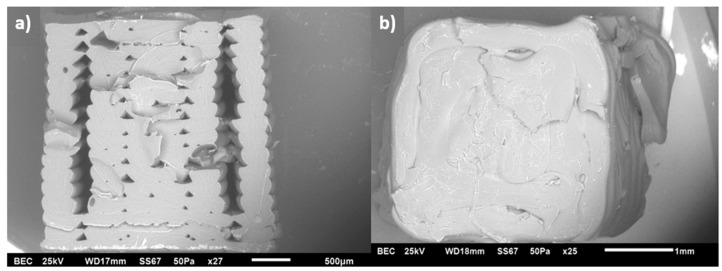
SEM images of MWCNT/ABS nanocomposites printed at (**a**) LBO and (**b**) TBO.

SEM images were taken to examine the microstructural features of FFF-printed specimens at 260 °C. Figure 10a,b show the structures of pure ABS specimens printed in two build orientations, LBO and TBO, respectively. The LBO specimen demonstrates good bonding between layers and fewer voids in the centre, while the TBO specimen exhibits a rougher surface morphology. 

**Figure 11:**
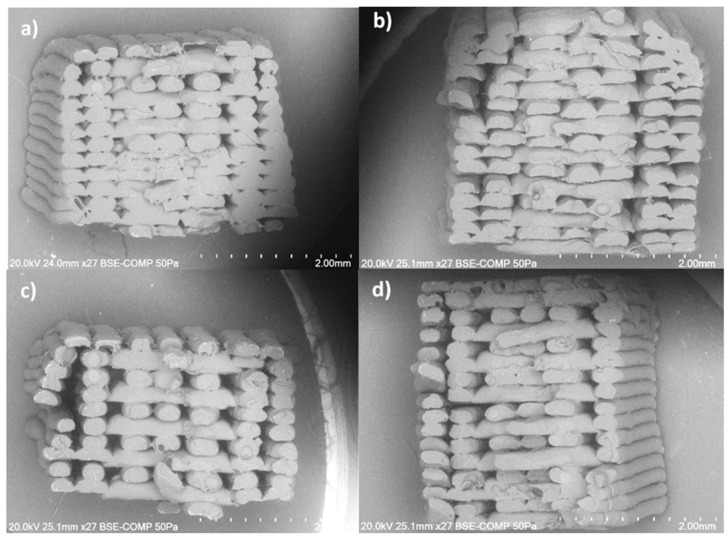
SEM images of MWCNT/ABS nanocomposites printed at LBO and 260 °C with (**a**) 0.1%, (**b**) 0.5%, (**c**) 1% and (**d**) 1.5% MWCNTs.

Figure 11 shows the FFF-printed nanocomposites at LBO with 0.1% to 1.5% MWCNTs inclusion and printed at 260 °C. By comparing the specimens of the pure ABS in Figure 10a, specimens with 0.1% and 1% nanoadditive percentages are missing the perimeters which were proved to have a crucial contribution to the overall strength in our previous work on the ABS polymer [40,41]. This indicates lower layer adhesion between the layers whereas the specimens with 0.5% and 1.5% nanoadditive percentages are intact where 0.5% shows less obvious air voids compared to that of the 1.5% specimens. These results also confirmed the adverse impact of the additives on 3D-printed structures as opposed to the upward trend frequently observed for the filament properties relative to the increase in additives [17]. This trend may be attributed to suboptimal reinforcement performance under different concentrations; (a) at the low concentration of 0.1%, due to insufficient CNT fraction; and (b) at the intermediate concentration of 1%, owing to CNT agglomeration that degrades dispersion and interfacial bonding. In contrast, the 0.5% concentration allowed optimal dispersion and interfacial contact, while the 1.5% concentration led to increased filler fraction and viscosity, promoting denser packing and improved bonding. Similar concentration-dependent trends have been observed in CNT–polymer systems, (a) where tensile strength peaks at optimal CNT content and declines when dispersion degrades [42]; (b) agglomeration effects are widely reported to lower composite performance [39]; and (c) the role of dispersion and interface in reinforcement efficacy is well-established [43].

**Figure 12:**
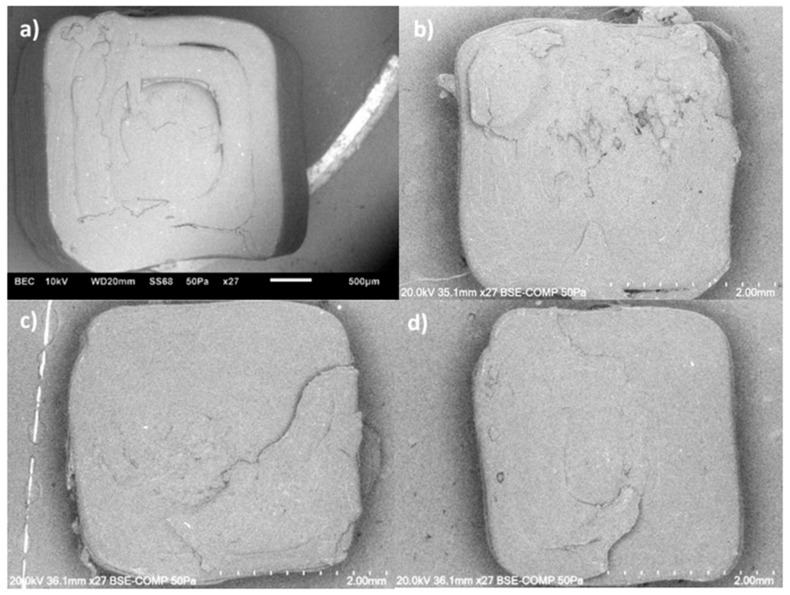
SEM images of MWCNT/ABS nanocomposites printed at TBO and 260 °C with (**a**) 0.1%, (**b**) 0.5%, (**c**) 1% and (**d**) 1.5% MWCNTs.

Figure 12 shows the SEM images of MWCNT/ABS nanocomposites at TBO with different nanoadditive percentages at a nozzle temperature of 260 °C. All specimens were printed with two contours as a fixed setting. While the overall fracture surfaces show only subtle differences, the 0.5% specimen appears to be slightly more uneven, which may correspond to improved interlayer bonding. The merging of the contour and infills in some images, such as Figure 12d, can be attributed to possible local printing irregularities. 

The obtained SEM images for FFF-printed specimens after fraction are in good agreement with the obtained tensile results, which can justify the reason why specimens with a 0.5% additive percentage are demonstrating better performance compared to the rest of the specimens in both LBO and TBO.

#### 3.1.5. Electrical Conductivity of Non-Functionalised MWCNT/ABS Nanocomposites

**Figure 13:**
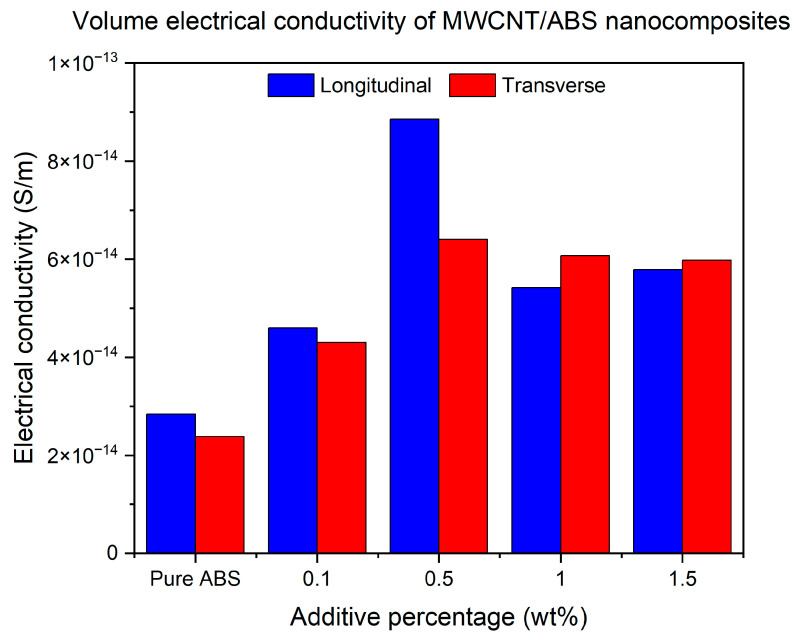
Volume electrical conductivity measurements for ABS specimens printed at LBO and TBO with different weight percentages of MWCNTs.

Figure 13 demonstrates the volume conductivity measurements for ABS specimens with different concentrations of MWCNTs. It is apparent that the reinforcement of MWCNTs as a nanofiller creates an effective electrical path that enhances the conductivity compared to the pure polymer [44]. Similarly to mechanical testing outcomes, the 0.5% additive percentage shows the maximum conductivity for both build orientations. The electrical conductivity measurements for LBO increase by 61%, more than 200%, 91% and 103% when the additive percentage increases from 0 to 1.5%. The specimens printed at TBO also show an increase of 80%, 170%, 155% and 151% when the additive percentage is 0.1%, 0.5% and 1.5%, respectively. The results are in good agreement with the literature as numerous studies reported electrical anisotropy when parts are printed at different angles [37]. Also, from the results, it can be concluded that the percolation threshold for the additive inclusion of MWCNTs in ABS happens at 0.5% as the electrical conductivity reaches its maximum at 0.5% and after that, a drop becomes almost constant at higher infill inclusions. The main reasons for this can be attributed to the dominance of air voids and the reduction in the interlayer bonding due to the agglomeration of additives [37]. This finding is similar to what was reported in the literature for MWCNT/ABS nanocomposites [45] as the electrical percolation threshold was found at around 0.6%.

The ANOVA for electrical conductivity in Table 5 did not reach statistical significance for additive loading or build orientation, reflecting the well-known high variability of CNT/polymer systems where conductive pathways depend sensitively on dispersion, agglomeration, and the formation of a percolated network. Nevertheless, the data still showed a practical optimum near 0.5% loading, consistent with percolation behaviour frequently reported for CNT–polymer composites. Variations in network connectivity and tunnelling contacts can mask significance at small sample sizes even when clear trends exist in mean values [46].

#### 3.1.6. Thermal Conductivity of Non-Functionalised MWCNT/ABS Nanocomposites

**Figure 14:**
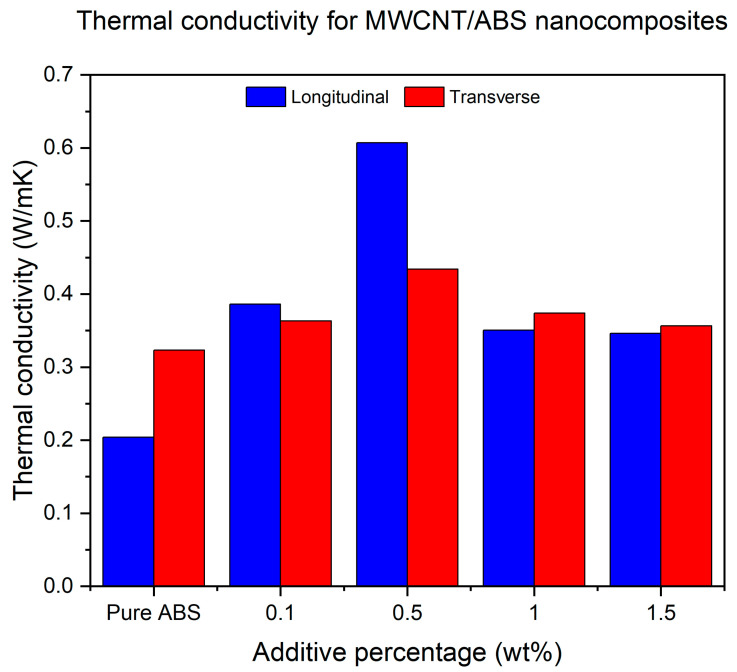
Thermal conductivity measurements for ABS specimens printed at LBO and TBO with different weight percentages of MWCNTs.

Similarly to the electrical conductivity measurements, the 0.5% MWCNT concentration in the ABS polymer shows the best thermal conductivity values among the rest of the batch. Figure 14 shows that the thermal conductivity value for LBO increases by almost 90% only by adding 0.1% of additive and then massively increases to almost 200% by adding 0.5% before decreasing to a third of that value for the specimens with 1 and 1.5% nanoadditive percentages. It is worth noting that by adding only 0.5% MWCNTs, the thermal conductivity of FFF-printed MWCNT/ABS nanocomposites printed at LBO is enhanced to 0.6 W/mK and is higher than their counterparts with only around 0.2 W/mK, despite being incorporated with 6% MWCNTs [16]. Moreover, it is worth noting that the incorporation of 0.5% MWCNTs in the current study yielded the superior thermal conductivity of 0.6 W/mK compared to the enhancement achieved with 10% MWCNT reinforcement in PEEK, which resulted in approximately 0.43 W/mK [19]. The transverse build orientation also increases by 12% by adding 0.1% of MWCNTs and then increases to 34% by adding 0.5% of the nanoadditives. This value decreases to 15% and 10% for 1% and 1.5% of MWCNT inclusion, respectively.

For the ANOVA of thermal conductivity in Table 6, the omnibus test was not significant, which can be attributed to measurement scatter dominated by microstructural features (voids, CNT distribution/alignment) and interfacial thermal resistance at CNT–polymer interfaces—which is well recognised as the primary bottleneck limiting composite k relative to intrinsic CNT values. Nonetheless, the literature consistently reports that improvements in thermal conductivity of CNT–polymer systems depend strongly on achieving uniform dispersion and effective interfacial coupling, where a balance between these factors governs phonon transport efficiency [47].

The interesting point to consider, which was common between both electrical and thermal conductivity investigations, was that the values of LBO are higher than TBO values until 0.5% of additive inclusion and then the transverse values proceed to the values of LBO. This could be occurring for two reasons. One is that because, at 0.5%, the layers are infused better than the other percentages (also confirmed by higher mechanical properties), therefore the electricity and heat were able to be transferred more easily through the layers. Secondly, it could be because, at higher nanoadditive percentages and TBO, the important factor in the transfer of heat and electricity is the amount of the additive that is available in the system. As the position of the filaments to the measuring sensor was parallel compared to the LBO; therefore, the electricity and heat were transferred a lot easier than in the LBO because the air voids are not the resisting factor for transferring the heat and electricity.

#### 3.1.7. Optimisation of Nanoadditives

From the previous sections, it was concluded that 0.5% MWCNTs in the ABS polymer optimises the thermo-electro-mechanical properties while demonstrating the most rigid structure. Therefore, functionalisation would be carried out for 0.5% MWCNTs to further improve the material properties of the MWCNT/ABS nanocomposites. The results of this process will be discussed in Section 3.2.

### 3.2. Investigation on the Properties of Functionalised MWNCT/ABS Nanocomposites

#### 3.2.1. FTIR, Raman Spectroscopy and SEM Images of Modified Additives

**Figure 15:**
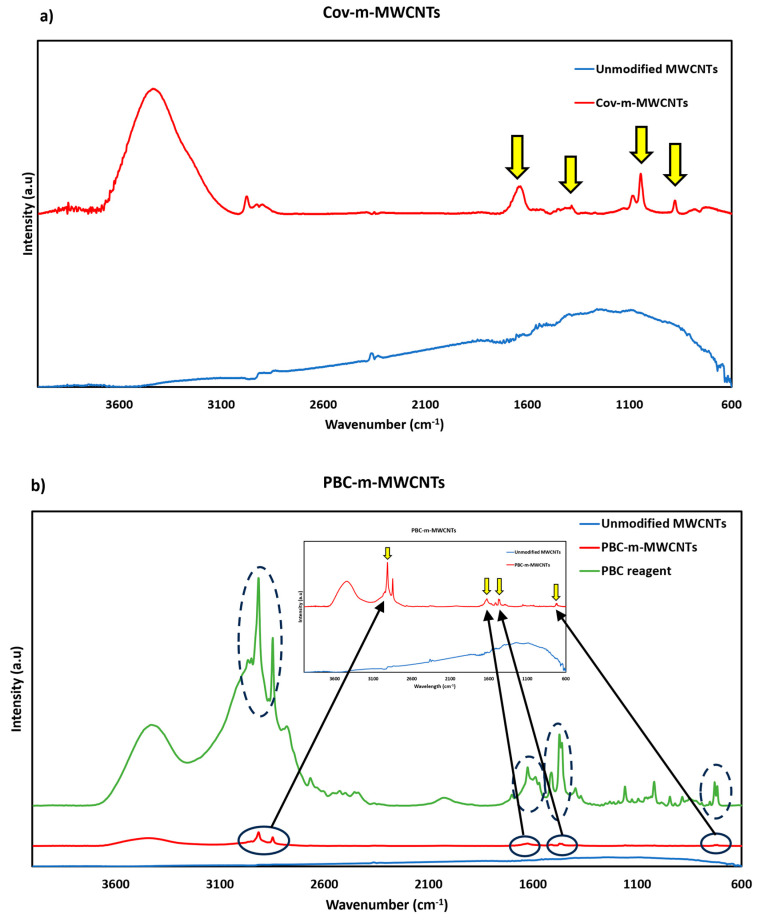
FTIR spectra of (**a**) Cov-m-MWCNTS and (**b**) PBC-m-MWCNTs. The black arrows indicate the correspondence between peaks in the main FTIR spectrum and their magnified view in the inset. The dashed lines/ovals highlight the spectral regions where characteristic peaks differ between samples.

To investigate the existence of the functional groups on the surface of the nanoadditives after the modification, FTIR and Raman spectroscopies were carried out. Figure 15 presents the FTIR results for MWCNTs modified covalently (Figure 15a) and non-covalently (Figure 15b). As can be seen, the FTIR spectrum of the pure and covalently functionalised MWCNTs shows some similar peaks that need to be addressed before highlighting the important peaks. The peaks at 2320 cm^−1^ and 2350 cm^−1^ are referred to as the backbone of the pristine MWCNT. Also, small humps at around 1540 cm^−1^ refer to the C=O stretch of carboxylic groups that were introduced during the purification procedure by HCL. The broad peak at around 2600 cm^−1^ to 3700 cm^−1^ are also characteristics of the O-H bonds and C-H vibrations. After modification, the important peaks were marked with yellow arrows for both covalent and non-covalent modifications. Functionalisation by strong acids in Figure 15a introduced four important peaks. The peak at 880 cm^−1^ refers to the C-H bending bonds, that between 1040 and 1100 cm^−1^ is related to the C-O bond type from alcohol and ether functional groups, the peak at around 1400 cm^−1^ refers to the carboxylic acid compound from the O-H bending group and the peak at around 1600 cm^−1^ is referred to as the C=O bond type from carboxyl and carbonyl groups [48].

In Figure 15b, the influence of the PBC reagent on the structure of the MWCNT can be seen with four major peaks when the green line (Pure PBC reagent) is compared with the red line (PBC modified MWCNT). The peaks at 2916 cm^−1^ and 2848 cm^−1^ are related to the C-H stretch in alkyl chains, and the peak at 1630 cm^−1^ is characteristic of the C=O bond type from carboxyl and carbonyl groups. The peaks between 1430 cm^−1^ and 1500 cm^−1^ are related to the O-H bond from carboxylic acid and the peak at around 730 cm^−1^ is the characteristic of the C-H bending bonds. Since the pristine MWCNT does not show any of the signals mentioned above and also the fact that the PBC-m-MWCNTs are adopting the same peaks as the ones in the PBC reagent, it shows that the PBC is attached to the surface of the MWCNTs with no damage to the functional groups [49].

**Figure 16:**
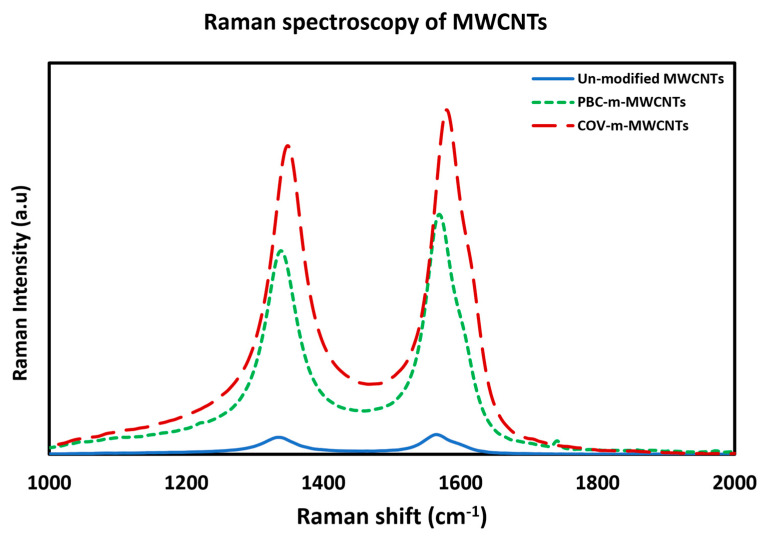
Raman spectra of pure MWCNT (blue line), Cov-m-MWCNT (red dashed line) and PBC-m-MWCNT (dashed green line).

To trace the changes in the structure and properties of the MWCNTs after modification and show which modification had less destruction to the structure of the MWCNTs, Raman spectroscopy was performed. Figure 16 shows the Raman spectroscopy results for the pure MWCNTs (blue line), PBC-m-MWCNTs (green dashed line) and Cov-m-MWCNTs (red dashed line). By calculating the ratio of the intensity of the D band over the G band (I _D/G_) for each batch, the pure MWCNTs have a value of 0.85 followed by 0.848 for PBC-m-MWCNTs and 0.9 for Cov-m-MWCNT specimens. This indicates that the non-covalent functionalisation has almost no effect on the structural integrity of the MWCNTs.

**Figure 17:**
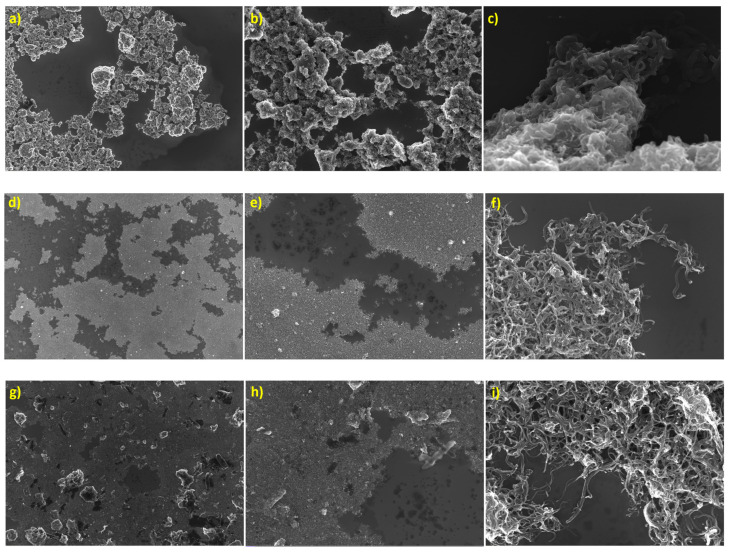
SEM images of (**a**–**c**) pure MWCNT, (**d**–**f**) Cov-m-MWCNTs and (**g**–**i**) PBC-m-MWCNT, all at 500×, 2000× and 50,000× magnifications, respectively.

The SEM images in Figure 17a–i also show the different magnifications of 500×, 2k× and 50k× for the pure MWCNT (Figure 17a–c), Cov-m-MWCNTs (Figure 17d–f) and PBC-m-MWCNTs (Figure 17g–i). As can be seen clearly from the photos, the agglomerated and entangled MWCNTs were de-agglomerated by both modification methods. The covalent modification has a better result in terms of separating the fibres from each other and dispersing them in the median. Similar SEM images showing the pattern of uniform dispersion were found when incorporating 6% of MWCNTs into the ABS matrix which later resulted in the highest elastic modulus [17]. The PBC modification still represents some agglomerated particles in the structure due to its moderate and non-destructive way of modification but still is considered a very efficient and successful method of modification.

#### 3.2.2. Mechanical Characterisation of Functionalised MWNCT/ABS Nanocomposites

The effects of covalent and non-covalent modification of MWCNT nanoparticles on the tensile strength (TS) and their associated root square error percentages (SQRT Error Mean) of the ABS polymer in the two longitudinal (LBO) and transverse (TBO) build orientations are listed in Table 7.

**Figure 18:**
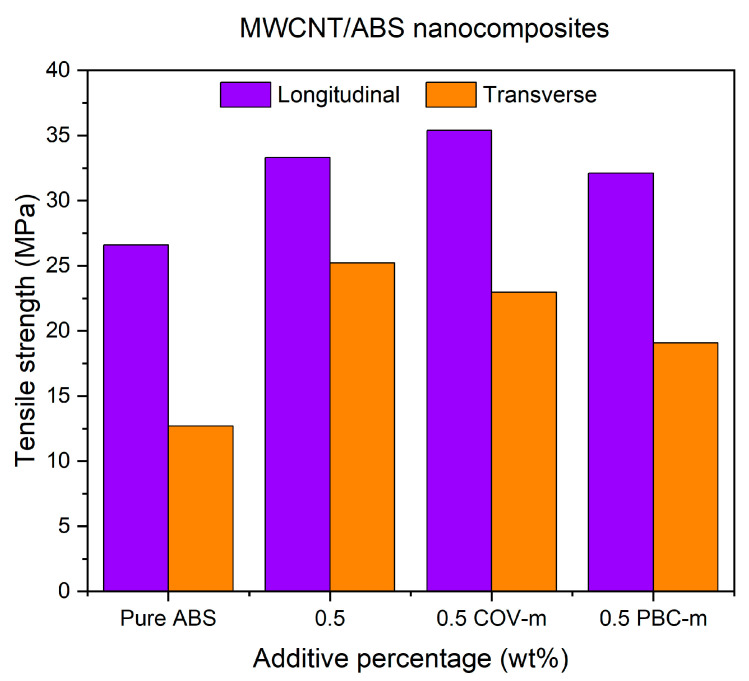
Tensile strength of functionalised MWCNT/ABS nanocomposites printed at LBO and TBO.

Figure 18 show the TS values for specimens at two build orientations. From the results, it can be concluded that the Cov-m-MWCNT specimens printed at LBO show the highest values while a slight decrease can be seen in specimens with PBC-m-MWCNTs. Although the specimens with Cov-m-MWCNT showed the best results at LBO, the TS values for the TBO were slightly lower than the unmodified specimens. It should also be mentioned that all composite specimens showed a lot better performance when compared with the pure ABS specimens.

These results can be explained by identifying differences in the interfacial mechanisms of the two functionalisation methods. Covalent modification creates a stronger chemical link between CNTs and the ABS matrix, not only enhancing stress transfer at LBO but also producing a stiffer interface that may promote brittle failure in TBO. In contrast, non-covalent PBC wrapping preserves the intrinsic CNT structure but reduces the effective CNT fraction, leading to a modest decrease in tensile strength. This trade-off between improved bonding and effective reinforcement content has been widely reported in CNT–polymer composites [27,39].

In addition, it should be noted that, due to the 1:1 mass ratio of PBC to CNTs used during the non-covalent functionalisation process, the effective CNT fraction in the PBC-m-MWCNT specimens was lower than that of the unmodified and covalently modified systems at the same nominal loading. This reduction in active CNT content likely contributed to the slightly lower tensile strength observed in the PBC-m-MWCNT composites. At the same time, the positive impact of PBC functionalisation on dispersion quality suggests that better interfacial distribution can partly compensate for the lower reinforcement fraction.

#### 3.2.3. Morphology Analysis of FFF-Printed Specimens

**Figure 19:**
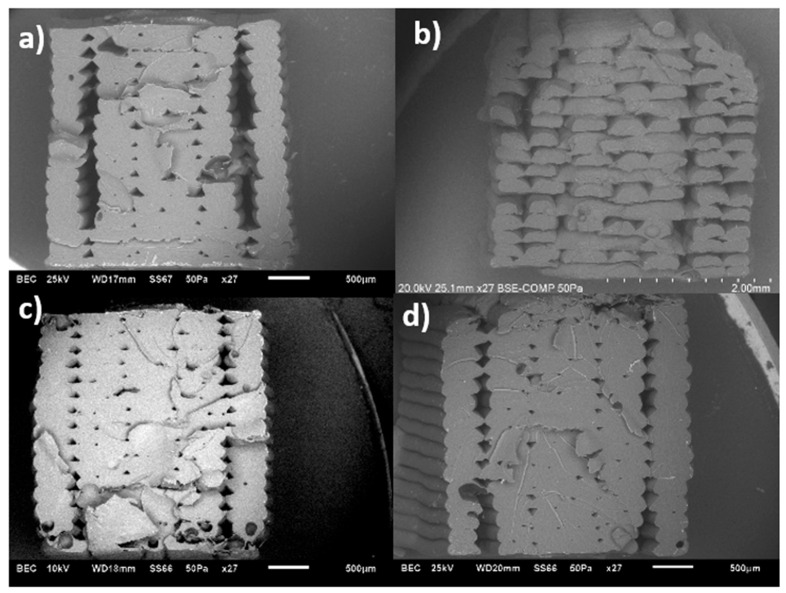
SEM images MWCNT/ABS nanocomposites printed at LBO and 260 °C with (**a**) 0.1%, (**b**) 0.5%, (**c**) 0.5% Cov-m and (**d**) 0.5% PBC-m MWCNTs.

SEM images from the fractured specimens built in LBO also confirm the obtained UTS values. From the images in Figure 19a–d, the nanocomposite specimens show layer delamination. When the modified additives are introduced, the layer delamination starts to decrease. With 0.5% of covalently modified MWCNTs, the fractured surface shows an uneven break pattern with fewer air voids and specimens with 0.5% PBC-m-MWCNTs also show an uneven fracture surface but with a few more air voids in the system.

**Figure 20:**
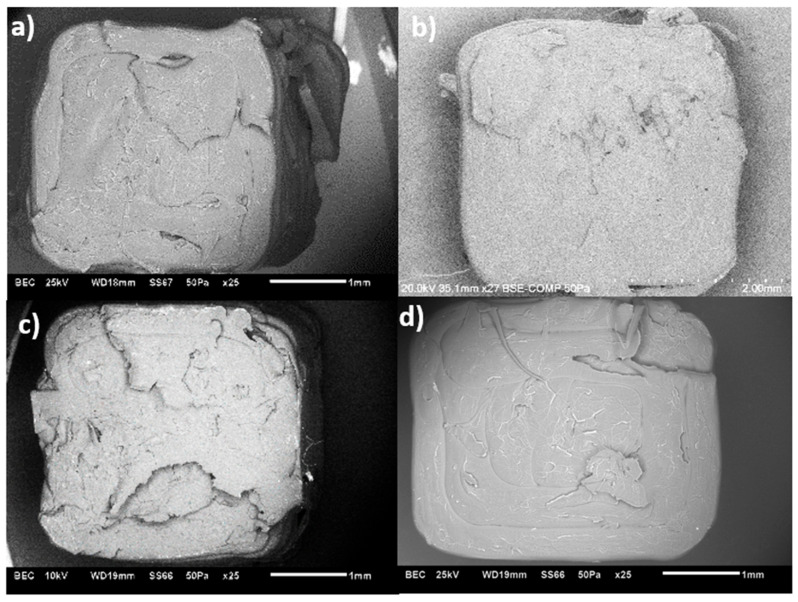
SEM images MWCNT/ABS nanocomposites printed at TBO and 260 °C with (**a**) 0.1%, (**b**) 0.5%, (**c**) 0.5% Cov-m and (**d**) 0.5% PBC-m MWCNTs.

When comparing the specimens printed in TBO (Figure 20a–d), at 260 °C, the unmodified specimen shows an uneven fracture surface with minimal air voids compared to the rest of the specimens.

#### 3.2.4. Electrical Conductivity of Functionalised MWNCT/ABS Nanocomposites

**Figure 21:**
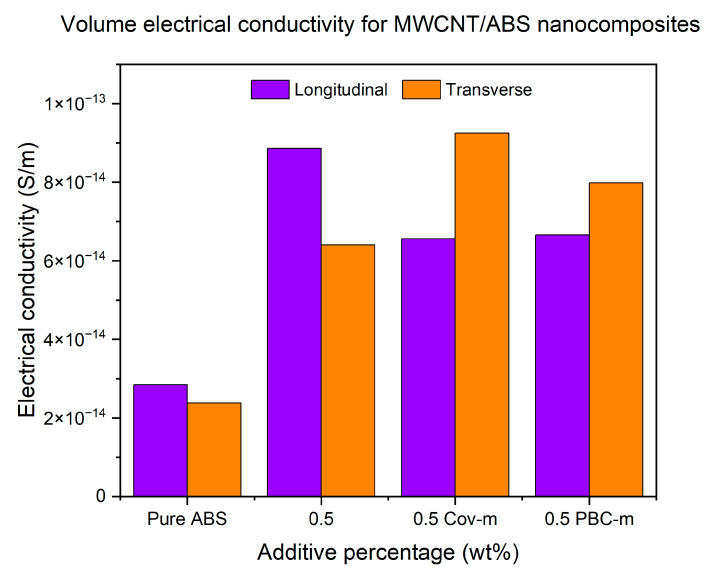
Volume electrical conductivity measurements for MWCNT/ABS specimens printed at LBO and TBO with modified and unmodified MWCNTs.

The volume electrical conductivity of the specimens is shown in Figure 21. The electrical conductivity for the specimens printed in LBO can be found at its highest when the nanoadditives were not modified. On the other hand, for the specimens printed at TBO, the electrical conductivity reaches its maximum when the Cov-m-MWCNTs were added to the system followed by the PBC-m-MWCNTs. However, the modified nanoadditives provided a positive impact on the TBO specimens with improvements of 45% and 25% for 0.5% Cov-m-MWCNTs and 0.5% PBC-m-MWCNTs, respectively, when compared with those with unmodified 0.5% MWCNTs.

This behaviour arises from the contrasting mechanisms of functionalisation. Unmodified MWCNTs preserve their pristine π-conjugation, enabling excellent electrical pathways in the longitudinal direction (LBO). However, at transverse orientation (TBO), where interlayer resistance is dominant, covalent functionalisation enhances interfacial adhesion and charge tunnelling across layers, thus improving conductivity. Conversely, non-covalent (PBC) functionalisation enhances dispersion without disrupting conjugation but may dilute the effective MWCNT content, leading to intermediate conductivity gains. Similar trade-offs between dispersion, interfacial conductivity, and conjugation integrity have been documented in MWCNT–polymer systems [50].

Moreover, the lower effective CNT fraction in the PBC-m-MWCNT specimens, due to the 1:1 mass ratio of PBC to CNTs, may also explain why their electrical conductivity at LBO was lower than that of the unmodified composites. Although PBC functionalisation improved dispersion and led to noticeable gains in TBO, the dilution of active CNT content reduced the overall number of conductive pathways. This observation is consistent with the mechanical results in Section 3.2.2, where a similar reduction in reinforcement efficiency was noted, and it reinforces the importance of balancing CNT fraction with functionalisation strategy to optimise multifunctional properties.

#### 3.2.5. Thermal Conductivity of Functionalised MWNCT/ABS Nanocomposites

**Figure 22:**
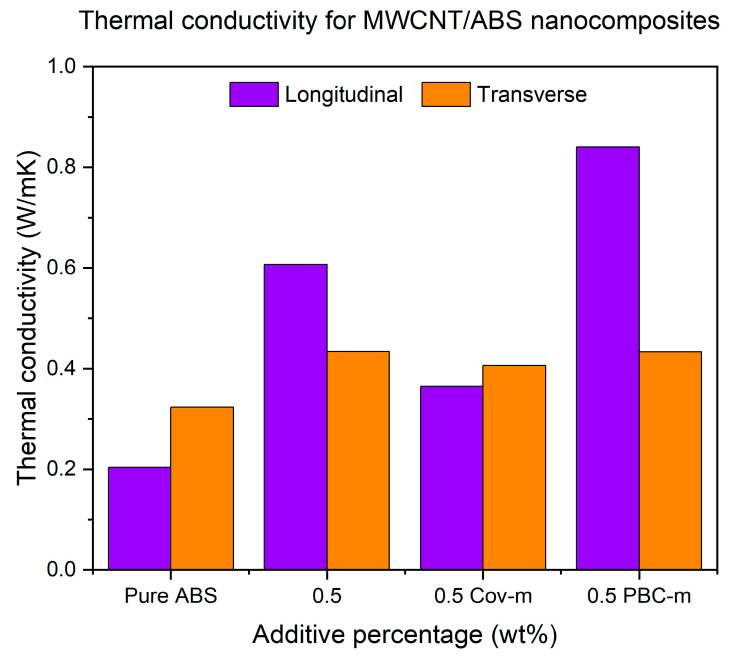
Thermal conductivity measurements for MWCNT/ABS specimens printed at LBO and TBO with modified and unmodified MWCNTs.

The thermal conductivity measurements for specimens with modified MWCNTs were carried out and the results are compared with the pure and unmodified specimens as shown in Figure 22. As it can be seen clearly, the unmodified specimens have an increase of 198% for LBO and 34% for TBO. However, the specimens with PBC-m-MWCNTs show a huge increase of almost 312% in thermal conductivity for LBO specimens and almost the same increase around 35% as the unmodified specimens for specimens printed in TBO. The Cov-m-MWCNTs also show an increase of 79% for LBO and 26% for TBO. This indicates that the modification with PBC has a very positive influence on the thermal conductivity properties of the MWCNT/ABS specimens.

These results highlight the critical role of interfacial structure in heat transport. While covalent functionalisation improves bonding, it introduces defects that scatter phonons and partially reduce thermal conductivity [51]. In contrast, non-covalent polymer wrapping preserves the intrinsic CNT lattice and π-conjugation, allowing more efficient phonon transmission while simultaneously improving dispersion [52]. This explains the exceptional increase observed with PBC-m-MWCNTs at LBO, where alignment facilitates conductive networks. Similar findings have been reported in CNT–polymer composites, where non-covalent strategies enhanced phonon transport while covalent routes often led to defect-induced scattering [53].

Moreover, the exceptional increase in thermal conductivity observed for PBC-m-MWCNT specimens is particularly noteworthy given that the effective CNT fraction was reduced by the 1:1 mass ratio of PBC to CNTs. This indicates that the improved phonon transport efficiency resulting from better dispersion and preserved conjugated structure in the non-covalent system outweighed the reduction in active CNT content.

### 3.3. Scalability and Industrial Challenges

Beyond the property enhancements demonstrated in this study, it is equally important to consider the practical challenges of translating these findings to industrial applications. While this study demonstrated the effectiveness of covalent and non-covalent functionalisation at laboratory scale, scalability challenges persist. Industrial adoption of functionalized MWCNT/ABS nanocomposites will require addressing the high CNT cost, maintaining consistent dispersion at production volumes, and adapting laboratory methods to continuous manufacturing. In addition, long-term stability and recyclability remain critical hurdles that must be evaluated for real-world applications. Prior reviews of CNT–polymer composites have emphasised these challenges and the need for cost-effective processing routes [54,55]. Future work will therefore focus on processing scale-up, ensuring dispersion quality, lifecycle cost assessments, and ageing performance under service conditions to enable wider adoption in commercial additive manufacturing.

## 4. Conclusions

In this research, MWCNT/ABS nanocomposites were first prepared at the 0.1%, 0.5%, 1% and 1.5% concentrations of MWCNTs and then FFF-printed specimens were fabricated for evaluating the effect of the MWCNTs on the mechanical, thermal and electrical properties of the 3D-printed nanocomposite. After determining the optimal content of MWCNTs as 0.5%, both covalent and non-covalent functionalisation of nanoadditives were performed to uniformly disperse the nanoadditives into the matrix and improve the interfacial bonding between them. Based on the research findings obtained from the experimental work, the following conclusions can be drawn:

- According to the experiment, FFF-printed MWCNT/ABS nanocomposites demonstrated better mechanical properties than pure ABS polymer. The presence of nanoadditives led to an increase in tensile strength for both specimens at longitudinal and transverse build orientations. The maximum tensile strength was found at 0.5% with an increase of 25% for LBO specimens and 98% for TBO specimens compared to pure polymer.
- Conductive MWCNTs were confirmed to generate an efficient electrical path in the ABS nanocomposites. The percolation threshold was found to be 0.5% which resulted in a 200% increase for LBO specimens and 170% for TBO specimens.
- Thermal conductivity was maximised at 0.5% MWCNTs for both build orientations. While LBO specimens illustrated a 200% increase in thermal conductivity, a 34% increase was observed for TBO specimens.
- According to SEM images, the presence of air voids, the agglomeration of nanoadditives and low interlayer bonding could be the main reasons for causing mechanical, electrical and thermal anisotropies.
- Generally, the functionalisation methods led to improvement in material properties when compared to the pure ABS polymer. For the tensile strength, improvements of 33% and 21% were found for LBO specimens with 0.5% Cov-m-MWCNTs and 0.5% PBC-m-MWCNTs, respectively. Regarding electrical properties, the functionalisation methods demonstrated a positive impact on TBO specimens with the enhancement of 45% and 25% for 0.5% Cov-m-MWCNTs and 0.5% PBC-m-MWCNTs, respectively, when compared with unmodified 0.5% MWCNTs. For the thermal conductivity, the best performance was recorded at 0.5% PBC-m-MWCNTs with a 312% increase compared to the pure ABS polymer.
- This study confirmed that while functionalisation markedly improves thermal and electrical conductivity through enhanced dispersion, its effect on tensile properties remains limited compared to the dominant influence of interlayer bonding and build orientation.

The conductive MWCNT/ABS nanocomposites with enhanced properties generated in this work can be widely adopted for applications across various industries such as aerospace, biomedical, and electronics industries. FFF-printed prototypes of efficient heat sinks and electrically conductive components using MWCNT/ABS nanocomposites can be direct products of future study.

## Figures and Tables

**Table 1:** Printing conditions for MWCNT/ABS nanocomposites.

Printing Process Settings	Value
Printing temperature	260 °C
Bed temperature	115 °C
Raster angle	±90°
Infill density	100%
Fill pattern	Rectilinear
Air Gap	0 mm
Printing Speed	70 mm/s
Layer thickness	0.2 mm
Raster width	0.5 mm
Nozzle diameter	0.4 mm
Build orientation	Longitudinal and Transverse

**Table 2:** TGA and DSC results of MWCNT/ABS nanocomposites.

Material	Tg (°C)	ΔCP (J/(g·K))	Decomposition Temperature (°C)	Residual Mass (%)
ABS polymer	111.1	0.20	408.9	3.91
ABS + 0.1% MWCNT	106.7	0.44	407.5	1.67
ABS + 0.5% MWCNT	107.2	0.39	407.8	3.21
ABS + 1% MWCNT	107.5	0.43	407.2	4.00
ABS + 1.5% MWCNT	107	0.44	407	3.60

**Table 3:** Tensile strength of MWCNT/ABS nanocomposites.

Tensile Strength (MPa)		
Nanoadditive Percentage	Longitudinal	Transverse
Pure ABS	26.6 ± 0.41	12.7 ± 1.37
0.1% MWCNT	32.4 ± 0.81	19.8 ± 0.89
0.5% MWCNT	33.3 ± 1.09	25.16 ± 0.41
1% MWCNT	27.8 ± 0.77	19.6 ± 0.53
1.5% MWCNT	32.4 ± 0.88	9.8 ± 0.23

**Table 4:** ANOVA of tensile strength.

Source	DF	Adj SS	Adj MS	F-Value	*p*-Value
Regression	2	431.831	215.915	8.19	0.015
AP	1	3.591	3.591	0.14	0.723
BO	1	428.239	428.239	16.24	0.005
Error	7	184.636	26.377		
Total	9	616.466			

**Table 5:** ANOVA of electrical conductivity.

Source	DF	Adj SS	Adj MS	F-Value	*p*-Value
Regression	2	0.000000	0.000000	1.31	0.328
AP	1	0.000000	0.000000	2.45	0.161
BO	1	0.000000	0.000000	0.17	0.690
Error	7	0.000000	0.000000		
Total	9	0.000000			

**Table 6:** ANOVA of thermal conductivity.

Source	DF	Adj SS	Adj MS	F-Value	*p*-Value
Regression	2	0.001041	0.000520	0.04	0.961
AP	1	0.000853	0.000853	0.07	0.804
BO	1	0.000188	0.000188	0.01	0.907
Error	7	0.090308	0.012901		
Total	9	0.091348			

**Table 7:** Tensile strength of MWCNT/ABS nanocomposites with modified nanoadditives.

Tensile Strength (MPa)		
Nanoadditive Percentage	Longitudinal	Transverse
Pure ABS	26.6 ± 0.41	12.7 ± 1.37
0.5% MWCNT	33.3 ± 1.09	25.2 ± 0.41
0.5% Cov-m-MWCNT	35.4 ± 0.39	23.0 ± 0.62
0.5% PBC-m MWCNT	32.1 ± 0.24	19.1 ± 0.14

## Data Availability

The original contributions presented in this study are included in the article. Further inquiries can be directed to the corresponding author.
